# Wet-chemical synthesis of two-dimensional metal nanomaterials for electrocatalysis

**DOI:** 10.1093/nsr/nwab142

**Published:** 2021-08-11

**Authors:** Zijian Li, Li Zhai, Yiyao Ge, Zhiqi Huang, Zhenyu Shi, Jiawei Liu, Wei Zhai, Jinzhe Liang, Hua Zhang

**Affiliations:** Department of Chemistry, City University of Hong Kong, Hong Kong, China; Department of Chemistry, City University of Hong Kong, Hong Kong, China; Hong Kong Branch of National Precious Metals Material Engineering Research Center (NPMM), City University of Hong Kong, Hong Kong, China; Department of Chemistry, City University of Hong Kong, Hong Kong, China; Department of Chemistry, City University of Hong Kong, Hong Kong, China; Department of Chemistry, City University of Hong Kong, Hong Kong, China; School of Materials Science and Engineering, Nanyang Technological University, Singapore 639665, Singapore; Department of Chemistry, City University of Hong Kong, Hong Kong, China; Department of Chemistry, City University of Hong Kong, Hong Kong, China; Department of Chemistry, City University of Hong Kong, Hong Kong, China; Hong Kong Branch of National Precious Metals Material Engineering Research Center (NPMM), City University of Hong Kong, Hong Kong, China; Shenzhen Research Institute, City University of Hong Kong, Shenzhen 518057, China

**Keywords:** two-dimensional, metal nanomaterials, wet-chemical synthesis, phase, heterophase, electrocatalysis

## Abstract

Two-dimensional (2D) metal nanomaterials have gained ever-growing research interest owing to their fascinating physicochemical properties and promising application, especially in the field of electrocatalysis. In this review, we briefly introduce the recent advances in wet-chemical synthesis of 2D metal nanomaterials. Subsequently, the catalytic performances of 2D metal nanomaterials in a variety of electrochemical reactions are illustrated. Finally, we summarize current challenges and highlight our perspectives on preparing high-performance 2D metal electrocatalysts.

## Teaser

This review article gives a brief overview and perspectives on recent research advances in the wet-chemical synthesis of various 2D metal nanomaterials for electrocatalytic applications.

## INTRODUCTION

With the increasingly serious global energy and environmental crisis caused by the excessive consumption of non-renewable fossil fuels, it is urgent to develop clean, efficient and sustainable energy conversion technologies [1,2]. As one of the most promising methods of producing clean energy, electrocatalysis can effectively convert incombustible molecules (e.g. water and carbon dioxide (CO_2_)) into renewable fuels, including hydrogen, carbon monoxide (CO), alcohols and hydrocarbons [3,4]. However, the major challenges in electrocatalysis are the sluggish kinetics and high overpotentials in electrochemical reactions, as well as the high cost and low stability of electrocatalysts [5–7]. Thus, rational design and preparation of highly efficient and stable catalysts in electrochemical energy conversion, such as fuel cells, metal-air batteries and water electrolysis, have become one of the most popular research directions [8–10].

Two-dimensional (2D) nanomaterials have been extensively studied in electrocatalysis due to their high surface-to-volume ratio, free dangling bonds and abundant active sites [11–13]. A vast library of novel 2D electrocatalysts has been synthesized, such as transition metal dichalcogenides (TMDs), black phosphorous, hexagonal boron nitride (*h*-BN), layered double hydroxides (LDHs), MXenes, polymers, metal-organic frameworks (MOFs), covalent-organic frameworks (COFs), metals and metal oxides [14–18]. In particular, 2D metal nanomaterials have gained ever-growing attention in electrocatalysis due to their high conductivity and superior catalytic activities [15,19].

As known, it is difficult for the intrinsically non-layered metals to spontaneously form 2D morphology, since 2D metallic nanostructures possess the fairly large surface energy of a specific facet, which is thermodynamically unfavorable [20]. As known, capping agents used in wet-chemical synthesis can greatly reduce the surface energy of nanomaterials and thus facilitate growth along specific crystalline orientations of metal nanocrystals [15]. Hence, the wet-chemical synthetic method using various kinds of capping agents has become the most powerful strategy for synthesizing 2D metal nanomaterials. In past decades, a library of 2D noble metal nanomaterials, such as Au [21], Ag [22], Pt [23] and Pd [24], have been successfully synthesized [25]. However, practical applications of 2D noble metal nanomaterials are severely hindered by the high cost and scarcity of noble metals [26]. Hence, tremendous effort has been devoted to the preparation of 2D non-noble metal nanomaterials and 2D metal alloys, which are then used as high-performance and cost-saving electrocatalysts.

**Figure 1.**
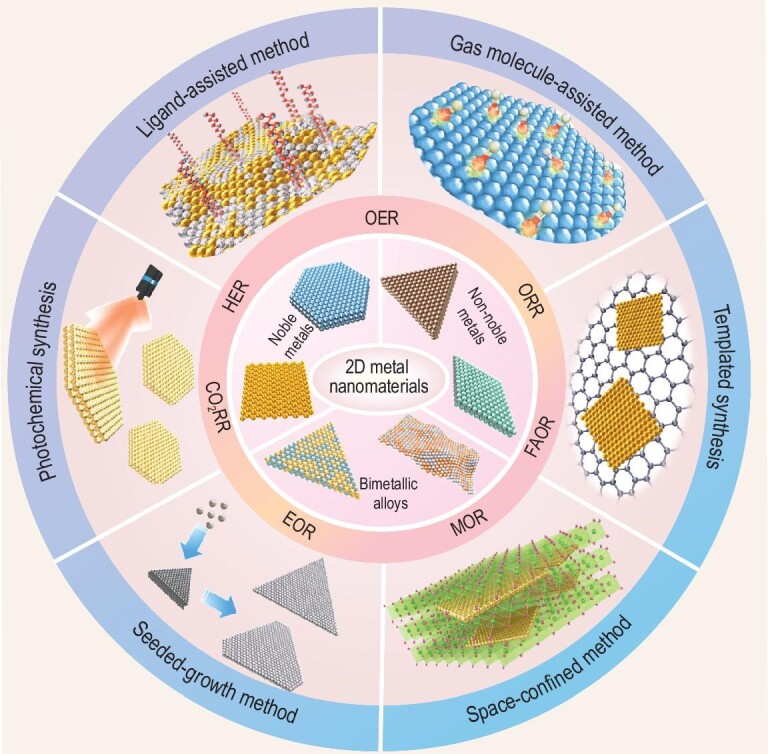
Schematic illustration of wet-chemical synthesis of various 2D metal nanomaterials for various electrocatalytic reactions.

Here, we give a brief overview of the recent research advances in the wet-chemical synthesis of various 2D metal nanomaterials and their application in electrocatalysis (Fig. 1). First, we introduce typical wet-chemical synthetic methods for the preparation of 2D metal nanomaterials (e.g. noble metals, non-noble metals and bimetallic alloys), such as ligand-assisted synthesis, gas-molecule-assisted synthesis, templated synthesis, space-confined synthesis and seeded-growth synthesis. Then, we summarize the electrocatalytic applications of 2D metal nanomaterials in the hydrogen evolution reaction (HER), oxygen reduction reaction (ORR), oxygen evolution reaction (OER), ethanol oxidation reaction (EOR), methanol oxidation reaction (MOR), formic acid oxidation reaction (FAOR) and carbon dioxide reduction reaction (CO_2_RR). Finally, challenges, as well as our personal perspectives on future research directions, are presented.

## SYNTHESIS OF 2D METAL NANOMATERIALS

The controlled synthesis of 2D metal nanomaterials with desired composition, size, thickness and crystal phase is essential for exploring their physicochemical properties and various applications. In this section, some representative wet-chemical methods for the synthesis of 2D metal nanomaterials are introduced. Table 1 summarizes some representative 2D metal nanomaterials prepared by various wet-chemical synthetic methods.

**Table 1.** Summary of some representative 2D metal nanomaterials prepared by various wet-chemical synthetic methods.

Synthetic method	Element	Morphology	Phase	Basal plane	Thickness	Lateral dimension	Ref.
							
Ligand-assisted	Au	Nanoribbon	4H	(110)	2–6 nm	Length: 0.5–6.0 μm	[27]
						Width: 15.0–61.0 nm	
Ligand-assisted	Au	Nanosheet	2H/*fcc*	(11$\bar{2}$0)_2H_/(101)*_fcc_*	∼8 nm	416 ± 160 nm	[32]
Ligand-assisted	Au	Triangular nanoplate	*fcc*	(111)	∼15 nm	∼45–120 nm	[43]
Ligand-assisted	Ag	Triangular nanoplate	*fcc*	(111)	∼11.3 nm	40 ± 120 nm	[39]
Ligand-assisted	Pt	Nanodendrite	*fcc*	(110)	∼2.3 nm	2.5 nm	[23]
Ligand-assisted	Pd	Nanosheet	*fcc*	(110)	∼0.8 nm	100–200 nm	[35]
Ligand-assisted	Pd	Square-like	*fcc*	(100)	∼2.5 nm	∼80 nm	[36]
		Irregular polygon		(110)		∼150 nm	
		Hexagonal sheet		(111)		∼100 nm	
Ligand-assisted	Cu	Triangular nanosheet	*fcc*	(111)	∼5 nm	∼1.7 ± 0.5 μm	[33]
Ligand-assisted	Co	Partially oxidized sheet	*hcp*	(001)	0.84 nm	∼500 nm	[29]
Ligand-assisted	Cd	Hexagonal nanosheet	*hcp*	(002)	30–50 nm	1–3 μm	[38]
Ligand-assisted	Sb	Hexagonal nanosheet	*rhombohedral*	(001)	5–30 nm	0.5 ± 1.5 μm	[44]
Ligand-assisted	PdAg	Nanodendrite	*fcc*	(110)	5–7 nm	∼80 nm	[34]
Ligand-assisted	Pd_3_Pb	Square nanosheet	*fcc*	(100)	∼5.2 nm	∼200 nm	[45]
Ligand-assisted	RuCu	Channel-rich snowflake-like nanosheet	*hcp/amorphous*	–	∼6 nm	∼80 nm	[30]
Ligand-assisted	RuCu	Lichen-like nanosheet	*hcp*	–	∼5 nm	∼30 nm	[31]
Ligand-assisted	PtPb	Hexagonal nanoplate	*hcp*	(001)	4.5 ± 0.6 nm	∼16 nm	[28]
Ligand-assisted	RuRh_2_	Nanoring	*fcc*	–	∼0.83 nm	–	[41]
Gas molecule-assisted	Pd	Hexagonal nanoplate	*fcc*	(111)	1.8 nm	60 nm	[24]
Gas molecule-assisted	Pd	Nanosheet	*a/c* ^a^	–	∼1.0 nm	–	[47]
Gas molecule-assisted	Pd	Nanosheet	*fcc*	(110)	3 ± 1 ML^b^	120–260 nm	[48]
					5 ± 1 ML	50–150 nm	
					8 ± 1 ML	∼20 nm	
Gas molecule-assisted	Pd	Nanomesh	*fcc*	–	∼3.3 nm	–	[49]
Gas molecule-assisted	Rh	Parallelogram nanosheet	*fcc*	(111)	0.9 ± 0.4 nm	Length: 880 nm	[50]
						Width: 450 nm	
Gas molecule-assisted	Rh	Nanosheet	–	–	∼0.4 nm	500–600 nm	[51]
Gas molecule-assisted	Rh	Nanosheet	*fcc*	–	∼1.2 nm	–	[52]
Gas molecule-assisted	Rh	Hierarchical nanosheet	*hcp*/VBP	(001)	3.7 ± 1.1 nm	21.4 ± 7.6 nm	[68]
Gas molecule-assisted	Ru	Triangular nanoplate	*hcp*	(0001)	3 ± 0.6 nm	23.8 ± 4.6 nm	[53]
		Irregular nanoplate			1.5 ± 0.2 nm	15.1 ± 2.7 nm	
Gas molecule-assisted	Ir	Mesoporous nanosheet	*fcc*	–	∼10 nm	–	[54]
Gas molecule-assisted	Ir	Partially hydroxylated nanosheet	*fcc*	(111)	∼1.3 nm	∼57 nm	[55]
Gas molecule-assisted	Al	Nanosheet	*fcc*	(111)	∼1.5 nm	200–600 nm	[69]
Gas molecule-assisted	PtBi	Hexagonal nanoplate	*fcc*	–	∼18 nm	∼100 nm	[56]
Gas molecule-assisted	PtCu	Nanosheet	*fcc*	(111)	∼1.6 nm	∼13 nm	[57]
Gas molecule-assisted	PdZn	Nanosheet	*fct*	–	∼3 nm	–	[58]
	PdCd				∼4.8 nm		
Gas molecule-assisted	PdCu	Nanosheet	*a/c*	(111)	∼1.2 nm	–	[59]
Gas molecule-assisted	PdCu	Nanosheet	*fcc*	(111)	2.8 ± 0.3 nm	–	[63]
Gas molecule-assisted	Pd_4_Cu_1_	Nanosheet	*fcc*	(111)	2.7 ± 0.5 nm	33.8 ± 8.3 nm	[61]
Gas molecule-assisted	PdMo	Bimetallene	*fcc*	(111)	0.88 nm	∼100 nm	[60]
Gas molecule-assisted	PdIr	Bimetallene	*fcc*	(111)	∼1 nm	20–30 nm	[62]
Gas molecule-assisted	RuNi	Quasi-hexagonal nanosheet	*hcp*	(0001)	11.6 ± 3.2 nm	80.7 ± 14.1 nm	[64]
Gas molecule-assisted	RuRh	Triangular nanosheet	*hcp*	(0002)	1.9 ± 0.5 nm	∼16.3 nm	[65]
Gas molecule-assisted	RhCu	Nanosheet	*a/c*	–	∼1.3 nm	–	[52]
	RhZn						
	RhRu						
Gas molecule-assisted	RhCo	Nanosheet	*fcc*	–	∼1.3 nm	–	[66]
Templated synthesis	Au	Square nanosheet	2H	(110)	∼2.4 nm	200–500 nm	[21]
Space-confined	Au	Nanosheet	*fcc*	(001)	∼1.0 nm	–	[79]
Space-confined	Au	Nanosheet	*fcc*	(111)	∼10 nm	∼3 μm	[82]
Space-confined	Pd	SAL	*fcc*	(111) & (200)	∼0.3 nm	5–50 nm	[80]
	PdCo						
Space-confined	Ag	Nanosheet	*fcc*	(111)	4.8 ± 0.3 nm	–	[81]
Space-confined	Rh	Porous nanosheet	*fcc*	(111)	∼1.7 nm	–	[83]
Seeded-growth	Au	Nanoplate	*fcc*	(111)	∼20 nm	50–500 nm	[85]
Seeded-growth	Ag	Triangular nanoplate	*fcc*	(111)	–	100–600 nm	[84]
Seeded-growth	Ag	Triangular nanoplate	*fcc*	(111)	5–200 nm	45 nm–5 μm	[22]
Self-assembly	Au	Seaweed-like nanosheet	*fcc*	(111)	∼0.47 nm	–	[87]
Self-assembly	Pd	Porous nanosheet	*fcc*	–	∼10 nm	∼2.5 μm	[88]
Self-assembly	Ru	Nanosheet	*hcp*	(001)	∼1.0–1.2 nm	–	[89]
Photochemical synthesis	Au	Triangular & hexagonal nanoprism	*fcc*	(111)	22.0 ± 0.4 nm	498 ± 68 nm	[90]
Solvothermal	Ni	Nanosheet	*fcc*	(111)	2.2 nm	–	[91]
Solvothermal	Ni_4_Mo	Nanosheet	*bct*	(001)	2.0–2.1 nm	–	[92]
Solvothermal	CoFe	Nanosheet	*fcc*	(111)	2.1–2.7 nm	–	[93]
	NiFe						
	NiCo						

^a^Amorphous/crystalline; ^b^monolayers.

### Ligand-assisted method

**Figure 2.**
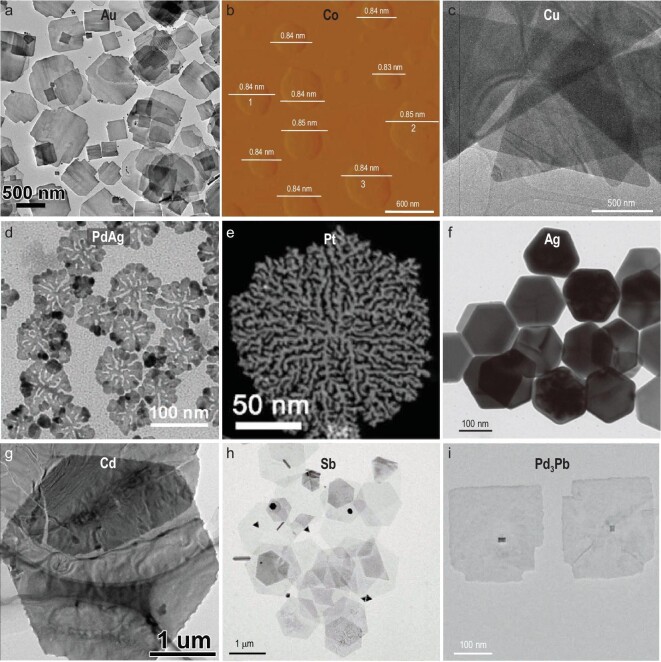
Ligand-assisted synthesis of 2D metal nanomaterials. (a) Transmission electron microscopy (TEM) image of freestanding 2H/*fcc* Au nanoplates. Reproduced with permission from ref. [32]. Copyright 2021 American Chemical Society. (b) AFM image of freestanding 4-atom-thick Co nanosheets. Reproduced with permission from ref. [29]. Copyright 2016 Nature Publishing Group. (c) TEM image of freestanding Cu nanosheets. Reproduced with permission from ref. [33]. Copyright 2019 Nature Publishing Group. (d) TEM image of 2D PdAg nanodendrites. Reproduced with permission from ref. [34]. Copyright 2018 John Wiley & Sons, Inc. (e) High-angle annular dark-field scanning-TEM (HAADF-STEM) image of 2D Pt nanodendrites. Reproduced with permission from ref. [23]. Copyright 2019 American Chemical Society. (f) TEM image of hexagonal Ag nanoplates. Reproduced with permission from ref. [37]. Copyright 2012 American Chemical Society. (g) TEM image of Cd nanosheets. Reproduced with permission from ref. [38]. Copyright 2016 John Wiley & Sons, Inc. (h) TEM image of freestanding Sb nanosheets. Reproduced with permission from ref. [44]. Copyright 2019 John Wiley & Sons, Inc. (i) TEM image of Pd_3_Pb square nanosheets. Reproduced with permission from ref. [45]. Copyright 2019 John Wiley & Sons, Inc.

Organic ligands, e.g. organic amines [27–32], halogenic-organic ligands [23,33–36] and polymeric ligands [37–41], have been widely used to synthesize 2D metal nanomaterials, and can not only lower the surface energy of their specific facets, but also stabilize their 2D structure [15]. For instance, our group first synthesized 4H-phase Au nanoribbons with a thickness of 2–6 nm using oleylamine and 1,2-dichloropropane as organic ligands in the presence of hexane. The use of 1,2-dichloropropane is critical to the formation of 2D morphology; without it, only Au nanoparticles were obtained [27]. Very recently, our group also prepared square-like, freestanding Au nanosheets with a mixture of 2H and face-centered cubic (*fcc*) crystal phase (Fig. 2a) through reducing HAuCl_4_·3H_2_O in a mixture of oleylamine, hexane, squalene, 1,2-dichlorobutane and 4-*tert*-butylpyridine [32]. The obtained 2H/*fcc* Au nanosheets possessed an edge length of 416 ± 160 nm, and a thickness of ∼8 nm. Impressively, the 2H/*fcc* Au nanosheets possessed a unique structure, i.e. 2H/*fcc* edges and 2H/*fcc* basal planes with *fcc*-stacking faults and/or twin boundaries, and *fcc* edges with few or no aforementioned defects. In addition, it has been demonstrated that the organic amines are able to coordinate with complex intermediates and form a ligand-confined space to guide the formation of 2D structures. For example, Gao *et al.* found that *n*-butylamine could specifically adsorb on the surface of [Co(H_2_O)_6_]^3+^ intermediates to efficiently reduce their surface energy and avoid their aggregation, leading to the formation of 2D Co nanosheets during the subsequent condensation process [29]. The average thickness of the obtained Co nanosheets, 0.84 nm, was confirmed by atomic force microscopy (AFM) (Fig. 2b), and is close to that of a 4-atom-thick Co sheet along the [001] direction. Besides the aforementioned hydrophobic organic ligands, various kinds of hydrophilic organic ligands can also be used to produce 2D metal nanomaterials in polar solvents. For instance, Luc *et al.* successfully synthesized freestanding Cu nanosheets (Fig. 2c) via reducing Cu(II) nitrate by _L_-ascorbic acid (_L_-AA) in the presence of cetyltrimethylammonium bromide (CTAB) and hexamethylenetetramine (HMTA) [33]. The _L_-AA not only reduced Cu^2+^, but also protected the obtained Cu nanosheets from oxidation. The use of CTAB and HMTA provided an alkaline environment to stabilize the Cu^+^, so that the disproportionation reaction was suppressed and growth kinetics were controlled to favor the formation of 2D morphology. Moreover, 2D snowflake-like PdAg alloy nanodendrites (Fig. 2d) were synthesized using octadecyltrimethylammonium chloride (OTAC) as the structure-directing agent [34]. The OTAC selectively passivated the {110} plane of PdAg nanodendrites (Fig. 2d) during the fast reduction process of Pd and Ag, thus leading to the formation of such ultrathin nanodendrites. Notably, Xu *et al.* prepared ultrathin Pd nanosheets through the confined growth inside lamellar micelles constructed by docosylpyridinium bromide [35]. In another study, the same group also synthesized ultrathin Pt nanodendrites (Fig. 2e) using a long-chain amphiphilic surfactant (C_22_H_45_-N^+^(CH_3_)_2_CH_2_COOH(Br^−^)) as the structure-directing template [23]. Additionally, polymeric ligands with super-long molecular chains are another category of widely used capping agents, and can reduce the reaction rate and effectively prevent the aggregation of nanostructures. For example, Kim *et al.* employed a combinatorial library of 24 polymers (10 cationic, 6 anionic and 8 neutral polymers) to synthesize Ag nanoplates (Fig. 2f) in an aqueous system [37]. Notably, neutral and anionic polymers tended to favor the formation of 2D Ag nanoplates, while the use of cationic polymers led to the formation of spheroidal Ag nanoparticles. Besides the negative and neutral charges, they found that the appropriate length and amphiphilic structure of polymers were the keys for the formation of Ag nanoplates. Among the various polymeric ligands, poly(vinylpyrrolidone) (PVP) has attracted great attention in the synthesis of 2D metal nanomaterials because it can not only serve as the stabilizer, but also work as the mild reducing agent to kinetically control their nucleation and growth rate. To synthesize triangular Ag nanoplates with a uniform size distribution, Wijaya *et al.* proposed a PVP-assisted wet-chemical reduction method [39]. PVP acted as not only a surfactant, but also a weak reducing agent during the synthesis. Impressively, after introducing polyacrylamide and acetonitrile, the yield of Ag nanoplates was increased. Additionally, 2D Cd nanosheets with a thickness of 30–50 nm (Fig. 2g) were synthesized using PVP as surfactant and diethylene glycol as solvent in low temperature [38].

Inorganic ligands, such as halide ions (e.g. Cl^−^, Br^−^ and I^−^), can guide the growth of nanostructures in specific directions, as they can strongly bond with the surface metal atoms and adsorb on the selective facets of metal nanocrystals, thus serving as capping agents to modulate the 2D morphology of metal nanostructures. For instance, Kim *et al.* synthesized 2D Ag nanoplates using halide ions, i.e. Cl^−^, Br^−^ and I^−^, as the shaping agents [42]. Particularly, the chemisorption strength of halide ions on the (100) Ag surfaces followed the order of Cl^−^ < Br^−^ < I^−^, resulting in the different thickness of Ag nanoplates. Likewise, Chen *et al.* prepared high-yield monodispersed triangular Au nanoplates in the presence of tri-iodide ions (I_3_^−^) [43]. Peng *et al.* synthesized hexagonal few-layer antimonene (Sb) nanosheets (Fig. 2h) in a solution phase, in which the Cl^−^ and dodecylthiol (DDT) acted as the shape-directing agents [44]. When using SbCl_3_-DDT as precursors, rhombohedral phase Sb nanosheets with a thickness of 5–30 nm were obtained. The Cl^−^ ions were believed to passivate the (001) basal planes of Sb nanosheets and favor the growth of 2D nanosheets. Moreover, the synthesis of intermetallic *fcc* Pd_3_Pb nanoplates (Fig. 2i) with the help of Br^−^ was reported by Wang *et al.* [45]. Specifically, due to the strong selective bonding on the (100) facets of *fcc* Pd_3_Pb, Br^−^ served as a shape-directing agent to favor the formation of square Pd_3_Pb nanoplates with the exposure of (100) facets.

### Gas-molecule-assisted method

**Figure 3.**
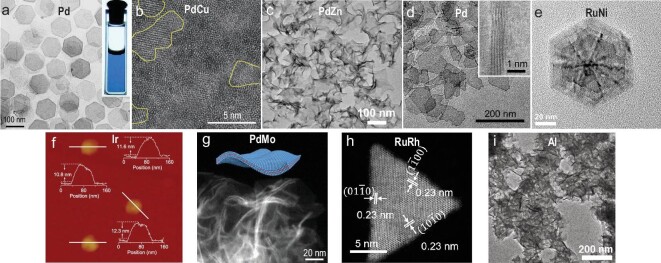
Gas molecular-assisted synthesis of 2D metal nanomaterials. (a) TEM image of freestanding ultrathin Pd nanosheets. Inset: photo of the dispersion of Pd nanosheets in ethanol. Reproduced with permission from ref. [24]. Copyright 2011 Nature Publishing Group. (b) High-resolution TEM (HRTEM) image of an amorphous/crystalline PdCu nanosheet. Yellow-curved areas show the crystalline parts of PdCu. Reproduced with permission from ref. [59]. Copyright 2019 Oxford University Press. (c) TEM image of PdZn nanosheets with *fct* phase. Reproduced with permission from ref. [58]. Copyright 2019 American Chemical Society. (d) TEM image of Pd nanosheets with 5 ± 1 Pd monolayers. Inset: side view of Pd nanosheets. Reproduced with permission from ref. [48]. Copyright 2019 American Association for the Advancement of Science. (e) HRTEM image of an individual RuNi nanostructure. Reproduced with permission from ref. [64]. Copyright 2019 Elsevier Ltd. (f) AFM image of freestanding Ir nanosheets. Reproduced with permission from ref. [55]. Copyright 2020 Oxford University Press. (g) HAADF-STEM image of freestanding PdMo bimetallenes. Inset: schematic illustration of PdMo bimetallene. Reproduced with permission from ref. [60]. Copyright 2019 Nature Publishing Group. (h) HAADF-STEM image of a single RuRh triangular nanosheet. Reproduced with permission from ref. [65]. Copyright 2020 Elsevier Inc. (i) TEM image of freestanding Al nanosheets. Reproduced with permission from ref. [69]. Copyright 2019 Elsevier Inc.

It has been found that gas molecules can play a significant role in the anisotropic growth of 2D metal nanomaterials. Among them, CO is the most extensively used as it can selectively adsorb on specific crystal facets of metals to form a strong bond with the surface atoms [46]. Such selective adsorption of CO significantly inhibits the growth of some specific facets of metal nanomaterials, resulting in the formation of 2D metals, including monometallic Pd [24,47–49], Rh [50–52], Ru [53] and Ir [54,55] and bimetallic alloy nanosheets based on Pt [56,57], Pd [58–63], Ru [64,65] and Rh [52,66]. In 2011, Huang *et al.* reported a CO-assisted method to synthesize hexagonal-shaped Pd nanosheets with a thickness of <10 atomic layers (Fig. 3a) by directly introducing 1 bar of CO gas to the aqueous solution [24]. The formation of 2D morphology of Pd nanosheets was ascribed to the inhibition of growth along the [111] direction due to the strong coordination of CO molecules with the (111) planes of *fcc* Pd. Similarly, the same group synthesized Rh nanosheets with few-atom-layer thickness by charging CO into the homogenous solution that consisted of rhodium(II) acetate, PVP and *N,N*-dimethylformamide (DMF) [50]. With the same strategy, Feng *et al.* also synthesized the intermetallic PtBi nanoplates with a regular hexagonal shape [56].

However, it is quite dangerous to directly use CO gas in reactions since CO gas is highly flammable and lethal. Therefore, many CO-releasing agents, including metal carbonyl compounds (e.g. molybdenum hexacarbonyl (Mo(CO)_6_), tungsten hexacarbonyl (W(CO)_6_)) [47,58–60,63], formaldehyde [51–53,64,66,67], formic acid [54,55] and DMF [54,56], are used to replace the CO gas. These compounds can decompose and release CO gradually at a certain elevated temperature. For instance, our group has recently reported a facile one-pot wet-chemical reduction method to synthesize ultrathin Pd nanosheets with the assistance of CO decomposed from Mo(CO)_6_ [47]. Importantly, the percentage of crystallinity could be tuned by changing the reaction temperature and a novel amorphous/crystalline heterophase could be obtained. With a similar strategy, our group also synthesized two types of ultrathin amorphous/crystalline heterophase PdCu nanosheets (Fig. 3b), i.e. amorphous phase-dominant nanosheets (*a*-PdCu) and crystalline phase-dominant nanosheets (*c*-PdCu) [59]. Interestingly, confirmed by Fourier transform infrared spectroscopy (FTIR), *a*-PdCu nanosheets adsorbed more CO groups than the *c*-PdCu after a 14-day aging process, resulting in different surface ligands and different crystallinities between *a*-PdCu and *c*-PdCu. Moreover, our group prepared a series of unconventional face-centered tetragonal (*fct*) PdM (M = Zn, Cd, ZnCd) nanosheets with a thickness of <5 nm (Fig. 3c) through heating palladium(II) acetylacetonate (Pd(acac)_2_), zinc(II) acetylacetonate (Zn(acac)_2_) and Mo(CO)_6_ in oleylamine [58]. It is worth mentioning that only PdZn nanoparticles with an irregular morphology were obtained under a similar condition without Mo(CO)_6_, indicating CO decomposed from Mo(CO)_6_ played a key role in the formation of 2D nanostructures. Remarkably, Wang *et al.* found that the thickness of Pd nanosheets (Fig. 3d) could be precisely controlled by using either pure CO gas or CO released from cobalt carbonyl (Co_2_(CO)_8_) [48]. Specifically, by precisely controlling the amount of CO gas and CO-releasing agent in synthetic reactions, Pd nanosheets with a thickness of 3 ± 1, 5 ± 1 and 8 ± 1 monolayers were prepared, respectively. In addition, some organics, such as formaldehyde and formic acid, could also release CO molecules during the synthetic process. For example, our group has synthesized the ultrathin flower-like Rh nanosheets with amorphous/crystalline heterophase in a diluted formaldehyde aqueous solution without any surfactant [52]. CO from the decomposition of formaldehyde adsorbed on the (111) facets of *fcc* Rh, leading to the formation of 2D morphology. The same synthetic strategy could also be applied to prepare a variety of amorphous/crystalline heterophase Rh-based bimetallic alloys (e.g. RhCu, RhZn and RhRu). Very recently, our group also employed formaldehyde to synthesize hierarchical Rh nanosheets with unconventional hexagonal close-packed (*hcp*) phase as well as novel ordered vacancies [68]. These highly voided, thermodynamically unfavorable 2D Rh nanostructures possessed a variety of vacated Barlow packings (VBPs) and orthorhombic symmetry, involving both perfect and vacated Rh nanosheets with diverse stacking sequences. In addition, Yin *et al.* rationally synthesized ultrathin *hcp* Ru triangle nanosheets from RuCl_3_ in the aqueous solution containing PVP and formaldehyde [53]. CO released from formaldehyde could selectively adsorb on the exposed (0001) planes of *hcp* Ru with low surface energies, resulting in the formation of ultrathin Ru nanosheets. Our group synthesized multilayered RuNi nanosheets with *hcp* phase (Fig. 3e) using benzyl alcohol as the solvent and formaldehyde as the reduction agent [64]. Cheng *et al.* reported a facile solvothermal method to synthesize the partially hydroxylated *fcc* Ir nanosheets with a thickness of 5–6 atomic layers (Fig. 3f) in the presence of iridium chloride, PVP, *N*-methylpyrrolidone and formic acid [55]. Mechanism study suggested that the formic acid played a crucial role in the formation of the sheet-like morphology, since CO released from formic acid could selectively bond to the (111) facets of Ir as a surface-confining agent.

Besides, the metal carbonyl precursors can not only serve as CO-releasing agents, but also act as metal precursors of desired alloys [60,62,65]. For example, Luo *et al.* prepared ultrathin *fcc* PdMo nanosheets with a sub-nanometer thickness of 0.88 nm (Fig. 3g) using Pd(acac)_2_ and Mo(CO)_6_ as metal precursors [60]. The study on the growth mechanism of PdMo nanosheets suggested that the small Pd nanosheets were first formed with the assistance of CO decomposed from Mo(CO)_6_, and then acted as seeds for the lateral growth and diffusion of Mo atoms. This approach could also be applied to synthesize PdW nanosheets after replacing Mo(CO)_6_ with W(CO)_6_. Moreover, serving as both metal precursors and CO-releasing agents, triruthenium dodecacarbonyl (Ru_3_(CO)_12_) [65] and tetrairidium dodecacarbonyl (Ir_4_(CO)_12_) [62] were used to synthesize the triangular *hcp* RuRh nanosheets with a thickness of 1.9 ± 0.5 nm (Fig. 3h) and ∼1 nm-thick *fcc* PdIr nanosheets, respectively, via a similar strategy.

Besides CO, oxygen (O_2_) could also act as the surface-confined agent to assist the formation of 2D metal nanostructures. For instance, Luo *et al.* developed a facile wet-chemical approach to preparing *fcc* Al nanosheets via reducing aluminum chloride in a non-protonic mesitylene solution under the atmosphere of N_2_ and O_2_ (Fig. 3i) [69]. Through tuning the concentration of O_2_ from 10 to 80 vol%, Al nanosheets with lateral sizes of 200–600 nm were obtained. The selective adsorption of O_2_ on the (111) facets of *fcc* Al was the key factor when forming such a 2D nanostructure. Impressively, the thickness of Al nanosheets could be finely tuned from 18 nm to sub-2 nm via increasing the ratio of O_2_ in the atmosphere of N_2_ and O_2_.

### Templated synthesis method

**Figure 4.**
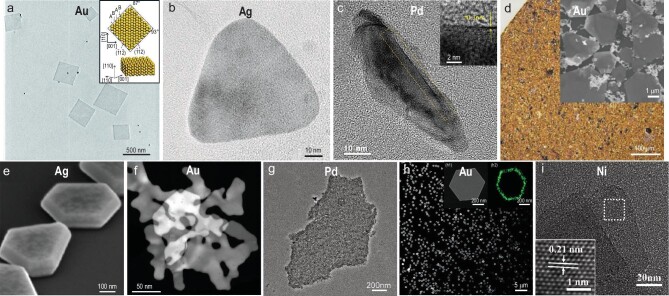
2D metal nanomaterials prepared by templated synthesis, space-confined method, seeded-growth method, self-assembly method, photochemical synthesis and solvothermal method. (a) TEM image of 2H Au nanosheets on graphene oxide template. Inset: schematic crystal structure of 2H Au. Reproduced with permission from ref. [21]. Copyright 2011 Nature Publishing Group. (b) TEM image of Ag nanoplate grown on MoS_2_ template. Reproduced with permission from ref. [70]. Copyright 2013 Nature Publishing Group. (c) TEM image of the side of a Pd SAL. Inset: HRTEM image of the yellow-curved area showing the single-atom thickness of the Pd SAL. Reproduced with permission from ref. [80]. Copyright 2019 Elsevier Inc. (d) Photo of Au nanosheets synthesized by space-confined method. Inset: scanning electron microscopy (SEM) image of space-confined Au nanosheets. Reproduced with permission from ref. [82]. Copyright 2020 Nature Publishing Group. (e) SEM image of Ag nanoplates synthesized by seeded-growth method. Reproduced with permission from ref. [22]. Copyright 2011 John Wiley & Sons, Inc. (f) HAADF-STEM image of Au nanosheets with seaweed-like morphology synthesized by self-assembly method. Reproduced with permission from ref. [87]. Copyright 2019 John Wiley & Sons, Inc. (g) TEM image of porous Pd nanosheets synthesized by self-assembly method. Reproduced with permission from ref. [88]. Copyright 2017 John Wiley & Sons, Inc. (h) SEM image of Au nanoprisms synthesized by plasmon-driven photochemical method. Insets: (h1) high-magnification SEM image of a single hexagonal Au nanoprism and (h2) nanoscale-secondary ion mass spectrometry image showing the elemental distribution of ^12^C^14^N^–^ signals (green) of the PVP ligands. Reproduced with permission from ref. [90]. Copyright 2016 Nature Publishing Group. (i) HRTEM image of Ni nanosheets. Inset shows the crystal lattices of Ni nanosheets. Reproduced with permission from ref. [91]. Copyright 2016 John Wiley & Sons, Inc.

Recently, various kinds of 2D materials, such as graphene oxide (GO), TMDs and hydroxides, have been applied as growth templates for the synthesis of 2D metal nanomaterials [21,70–73]. For instance, by using GO as a template, our group demonstrated the preparation of ultrathin 2D Au nanosheets with an unconventional 2H phase (Fig. 4a) [21]. Owing to the rich functional groups on GO, Au^3+^ could be adsorbed on the GO surface, and then mildly reduced into Au seeds by oleylamine. The Au seeds eventually assembled into ultrathin 2D square-like nanosheets with a thickness of ∼2.4 nm. Impressively, this is the first time Au nanosheets have been directly prepared with pure 2H phase through a wet-chemical route [74,75]. It is worth mentioning that the Au square-like nanoplates with an alternating 2H and *fcc* crystal phase could be obtained using the aforementioned 2H Au nanosheets as templates via secondary growth of Au [76]. Moreover, TMDs [77], one of the emerging 2D materials, can also serve as growth templates for a variety of noble metals. For example, our group has reported the epitaxial growth of Ag nanoplates on electrochemically exfoliated MoS_2_ nanosheets [78], in aqueous solution (Fig. 4b) [70]. Impressively, it was found that the growth of Ag nanoplates on MoS_2_ was oriented and the epitaxial relationship was 1/3{422}_Ag_||{100}_MoS2_. Additionally, hydroxides are also considered as ideal templates for the preparation of 2D metal nanomaterials. For instance, Dai *et al.* first prepared Ni_1−χ_Cu_χ_(OH)_2_ nanosheets as templates through substituting Ni^2+^ in the pre-synthesized Ni(OH)_2_ with Cu^2+^ [71]. Then the Cu^2+^ in Ni_1−χ_Cu_χ_(OH)_2_ nanosheets was reduced by DMF to obtain Cu nanosheets on the Ni(OH)_2_ templates in the presence of sodium formate. Moreover, after selectively etching the Ni(OH)_2_ nanosheets in a mixed solution of formic acid and sodium formate, freestanding Cu nanosheets were obtained.

### Space-confined method

Layered materials with interlayered 2D space can be used as space-confined templates to slow down the diffusion of metal ions and constrain the growth of metals into 2D morphology. For example, Wang *et al.* prepared ultrathin *fcc* Au nanosheets between the interlayers in the Mg-Al-LDHs. The Mg-Al-LDHs could not only effectively confine the crystal growth of 2D Au, but also stabilize the obtained Au nanosheets [79]. Moreover, Jiang *et al.* employed the layered crystalline clay mineral montmorillonite (MMT) as a template to prepare *fcc* single-atom-layer (SAL) Pd nanosheets (Fig. 4c) in the angstrom-sized interlayer space of MMT [80]. Impressively, freestanding Pd SAL was successfully prepared after the removal of MMT by acid etching.

Besides the aforementioned *hard* templates, the interfaces of liquid/gas and liquid/liquid phases can also serve as *soft* templates to confine the growth of 2D metal nanomaterials. For instance, Lee *et al.* demonstrated a redox-active peptide template-assisted method to synthesize multilayered single-crystalline *fcc* Ag nanosheets. In their reaction, a tyrosine-rich α-helical peptide was used to reduce the Ag ions at the water/air interface, resulting in the formation of single-crystalline Ag nanosheets [81]. Yue *et al.* utilized the dodecenylsuccinic acid bilayers as the *soft* template for the controlled growth of {111}-oriented *fcc* Au nanosheets (Fig. 4d) at the liquid/liquid interface [82]. Zhu *et al.* developed a 1-hydroxyethylidene-1, 1-diphosphonic-acid (HEDP)-mediated method to prepare hierarchical porous Rh nanosheets at the interface between the upper-layer solution phase and the bottom-layer emulsion phase [83].

### Seeded-growth method

The seeded-growth method represents a facile strategy to synthesize metal nanostructures with well-defined 2D morphology. It contains two steps, i.e. the pre-synthesis of seeds and the subsequent growth process. Generally, the lattice defects, e.g. twin boundaries, in a metal seed are more active for the heterogeneous nucleation, leading to preferential growth along the twin planes to form a sheet-like shape [22]. For instance, Khan *et al.* demonstrated the synthesis of large triangular Ag nanoplates from small Ag spherical seeds, and investigated the effect of poly(sodium 4-styrene-sulfonate) (PSSS) on regulating the shape of Ag [84]. The usage of PSSS generated the defective structures in Ag spherical seeds, which led to the anisotropic growth of Ag on the Ag seeds to form a 2D structure. Moreover, the kinetics control in the secondary growth of metal also plays an important role in directing the final shape of metal nanostructures when the defective metal nanocrystals are used as seeds. For example, Lin *et al.* employed defect-rich Au nanoparticles as seeds to direct the anisotropic growth of 2D Au nanoplates. The yield of Au nanoplates can be increased from 32.0% to 96.8% through tuning the molar ratio of PVP to HAuCl_4_ during the growth process [85]. In addition, Zeng *et al.* elaborated that the triangular Ag seeds could grow into either thin Ag nanoplates in a lateral-growth-favorable mode or thick Ag nanoplates in a vertical-growth-favorable mode (Fig. 4e) by using two different capping agents, i.e. sodium citrate and PVP, respectively [22].

### Other wet-chemical methods

Besides the aforementioned wet-chemical synthetic methods, other unique strategies have also been used to prepare 2D metal nanomaterials. For instance, our group utilized the ligand-exchange method to induce phase transformation from pre-synthesized 2H Au square sheets to *fcc* Au square sheets [86]. A similar crystal phase transformation from 4H Au nanoribbons to *fcc* Au nanoribbons was also observed [27]. Moreover, with the use of methyl orange, Ye *et al.* successfully synthesized the two-atomic-layer-thick 2D Au nanosheets with seaweed-like morphology (Fig. 4f), obtained via the self-assembly of small Au nanoflakes based on the oriented attachment mechanism [87]. Qiu *et al.* demonstrated a facile self-assembly approach for the synthesis of 2D porous *fcc* Pd nanosheets (Fig. 4g). Time-dependent experiments confirmed that the tiny Pd nanoparticles formed first, and gradually self-assembled into ultrathin one-dimensional nanowires with wavy orientation. Then, numerous ultrathin nanowires interweaved together and eventually formed porous Pd nanosheets [88]. Likewise, the oriented attachment assembly could also lead to the formation of Ru nanosheets [89]. Photochemical synthesis is another powerful strategy to prepare 2D metal nanostructures. For instance, the plasmon-driven synthesis of Au nanoprisms with a high yield of ∼90% has been achieved (Fig. 4h) through reducing HAuCl_4_ in the presence of PVP and methanol under visible light irradiation [90]. PVP not only acted as the surfactant, but also served as a photochemical relay to direct the anisotropic growth of Au nanoprisms. Moreover, Kuang *et al.* utilized Ni(OH)_2_, ethylene glycol and NaOH to synthesize single-crystalline ultrathin *fcc* Ni nanosheets (Fig. 4i) via solvothermal method [91]. With a similar strategy, NiMo, NiFe and NiCo nanosheets were also prepared [92,93].

## APPLICATION OF 2D METAL NANOMATERIALS IN ELECTROCATALYSIS

Metal nanomaterials are widely used as catalysts due to their superior activity and excellent stability in various electrochemical reactions. Recently, 2D metal nanomaterials have aroused tremendous research interest owing to their structural merits, including high surface-to-volume ratio, abundantly exposed surface active sites and unique electronic property. Here, as shown in Table 2, we summarize the recent progress in applying 2D metal nanomaterials as electrocatalysts in various electrochemical reactions, including water splitting (HER and OER), ORR, chemical fuel oxidation reactions (FAOR, MOR and EOR) and CO_2_RR.

**Table 2.** Summary of some 2D metal nanomaterials as electrocatalysts in various electrochemical reactions.

Application	Material	Phase	Electrolyte	Electrocatalytic performance	Ref.
					
HER	Pt nanodendrites	*fcc*	0.5 M H_2_SO_4_	η _HER_ <10 mV @ 50 mA cm^−2^	[23]
HER	{100}-exposed Pd nanosheets	*fcc*	0.5 M H_2_SO_4_	η _HER_ = 67 mV @ 10 mA cm^−2^	[36]
HER/OER	Partially hydroxylated Ir nanosheets	*fcc*	0.5 M H_2_SO_4_	η _HER_ ≈ 20 mV @ 10 mA cm^−2^	[55]
				η _OER_ = 328 mV @ 10 mA cm^−2^	
				η _HER_ ≈ 60 mV @ 10 mA cm^−2^	
			1.0 M KOH	η _OER_ = 266 mV @ 10 mA cm^−2^	
HER	Rh nanosheets	*fcc*	1.0 M KOH	η _HER_ = 42 mV @ 10 mA cm^−2^	[67]
HER	Hierarchical Rh nanosheets	*hcp*/VBP	1.0 M KOH	η _HER _= 37.8 mV @ 10 mA cm^−2^	[68]
HER/OER	Ru nanosheets	*hcp*	0.5 M H_2_SO_4_	η _HER_ = 20 mV @ 10 mA cm^−2^	[89]
				η _OER_ = 260 mV @ 10 mA cm^−2^	
HER/OER	IrRh nanosheet assemblies	*fcc*	1.0 M KOH	η _HER_ = 35 mV @ 10 mA cm^−2^	[40]
				η _OER_ = 251 mV @ 10 mA cm^−2^	
HER	RuRh_2_ bimetallene	*fcc*	0.5 M H_2_SO_4_	η _HER_ = 34 mV @ 10 mA cm^−2^	[41]
			1.0 M KOH	η _HER_ = 24 mV @ 10 mA cm^−2^	
			1.0 M PBS^a^	η _HER_ = 12 mV @ 10 mA cm^−2^	
HER/OER	RuCu snowflake-like nanosheets	*hcp/amorphous*	0.5 M H_2_SO_4_	η _HER_ = 19 mV @ 10 mA cm^−2^	[30]
				η _OER_ = 236 mV @ 10 mA cm^−2^	
			1.0 M KOH	η _HER_ = 20 mV @ 10 mA cm^−2^	
				η _OER_ = 234 mV @ 10 mA cm^−2^	
HER	RuNi nanostructures	*hcp*	1.0 M KOH	η _HER_ = 15 mV @ 10 mA cm^−2^	[64]
HER/OER	RhCo nanosheet aggregates	*fcc*	0.5 M H_2_SO_4_	η _HER_ = 12 mV @ 10 mA cm^−2^	[66]
				η _OER_ ≈ 250 mV @ 10 mA cm^−2^	
			1.0 M KOH	η _HER_ = 32 mV @ 10 mA cm^−2^	
				η _OER_ ≈ 240 mV @ 10 mA cm^−2^	
			1.0 M PBS	η _HER_ = 31 mV @ 10 mA cm^−2^	
				η _OER_ = 310 mV @ 10 mA cm^−2^	
HER	Ni_4_Mo nanosheets	*bct*	1.0 M KOH	η _HER_ = 35 mV @ 10 mA cm^−2^	[92]
HER/OER	CoFe nanosheets	*fcc*	1.0 M KOH	η _HER_ ≈ 30 mV @ 10 mA cm^−2^	[93]
				η _OER_ = 200 mV @ 10 mA cm^−2^	
OER	Mesoporous Ir nanosheets	*fcc*	0.5 M H_2_SO_4_	η _OER_ = 240 mV @ 10 mA cm^−2^	[54]
ORR	Porous Pt nanosheets	*fcc*	0.1 M HClO_4_	Mass activity: 2.07 A mg_Pt_^−1^	[96]
				Specific activity: 3.1 mA cm^−2^	
				@ 0.90 V (vs. RHE)	
ORR	PtPb/Pt nanoplates	*hcp/cubic*	0.1 M HClO_4_	Mass activity: 4.3 A mg_Pt_^−1^	[28]
				Specific activity: 7.8 mA cm^−2^	
				@ 0.90 V (vs. RHE)	
ORR	Intermetallic PtBi nanosheets	*hcp*	0.1 M HClO_4_	Mass activity: 1.04 A mg_Pt_^−1^	[56]
				@ 0.85 V (vs. RHE)	
ORR	5 monolayers Pd nanosheets	*fcc*	0.1 M KOH	Mass activity: 0.50 A mg_Pt_^−1^	[48]
				Specific activity: 0.70 mA cm^−2^	
			0.1 M HClO_4_	Mass activity: 0.30 A mg_Pt_^−1^	
				Specific activity: 0.42 mA cm^−2^	
				@ 0.95 V (vs. RHE)	
ORR	PdMo bimetallene	*fcc*	0.1 M KOH	Mass activity: 16.37 A mg_Pt_^−1^	[60]
				Specific activity: 11.64 mA cm^−2^	
				@ 0.90 V (vs. RHE)	
ORR	PdCo SAL nanosheets	*fcc*	0.1 M HClO_4_	Mass activity: 0.995 A mg_Pt_^−1^	[80]
				Specific activity: 0.343 mA cm^−2^	
				@ 0.90 V (vs. RHE)	
FAOR	Pd nanosheets	*fcc*	0.5 M H_2_SO_4 _+_ _0.25 M HCOOH	Mass activity: 1.38 A mg^−1^	[24]
				@ 0.14 V (vs. SCE^b^)	
FAOR	PdIr bimetallene	*fcc*	0.1 M HClO_4 _+_ _0.5 M HCOOH	Mass activity: 2.70 A mg _Pd+Ir_^−1^	[62]
				@ 0.50 V (vs. RHE)	
FAOR	Pd nanosheets	*fcc*	0.1 M HClO_4 _+_ _0.2 M HCOOH	Mass activity: 0.634 A mg_Pd_^−1^	[97]
				@ 0.68 V (vs. RHE)	
FAOR	PtPd nanosheets	*fcc*	0.5 M H_2_SO_4 _+_ _0.25 M HCOOH	Mass activity: 1.831 A mg_metal_^−1^	[98]
				@ 0.33 V (vs. RHE)	
FAOR	Porous Pd nanosheets	*fcc*	0.5 M H_2_SO_4 _+_ _0.5 M HCOOH	Mass activity: 0.409 A mg_metal_^−1^	[88]
				@ 0.18 V (vs. SCE)	
FAOR	PdCu nanosheets	*fcc*	0.5 M H_2_SO_4 _+_ _0.25 M HCOOH	Mass activity: 1.656 A mg_Pd_^−1^	[63]
				@ 0.12 V (vs. Ag/AgCl)	
MOR	Hierarchical porous Rh nanosheets	*fcc*	1 M KOH_ _+_ _0.5 M CH_3_OH	Mass activity: 0.333 A mg^−1^	[83]
				@ 0.63 V (vs. RHE)	
MOR	PtPb nanoplates	*a*/*c*	0.1 M HClO_4_ + 0.1 M CH_3_OH	Mass activity: 1.31 A mg^−1^	[99]
				Specific activity: 4.32 mA cm^−2^	
				@ 0.65 V (vs. RHE)	
MOR	Pd_4_Cu_1_ nanoplates	*fcc*	1 M KOH + 1 M CH_3_OH	Mass activity: 4.875 A mg^−1^	[61]
				@ 0.65 V (vs. RHE)	
EOR	Pd nanomeshes	*fcc*	1 M KOH + 1 M C_2_H_5_OH	Mass activity: 5.40 A mg_Pd_^−1^	[49]
				Specific activity: 7.09 mA cm^−2^	
				@ 0.80 V (vs. RHE)	
EOR	PdAg nanodendrites	*fcc*	1 M KOH + 1 M C_2_H_5_OH	Mass activity: 2.600 A mg^−1^	[34]
				@ −0.18 V (vs. SCE)	
EOR	PdZn nanosheets	*fct*	1 M NaOH + 1 M C_2_H_5_OH	Mass activity: 2.73 A mg_Pd_^−1^	[58]
				@ −0.20 V (vs. Ag/AgCl)	
CO_2_RR	Triangular Ag nanoplates	*fcc*	0.1 M KHCO_3_	96.8% FE for CO	[101]
				@ −0.855 V (vs. RHE)	
CO_2_RR	Au nanoribbons	4H	0.1 M KHCO_3_	90% FE for CO	[109]
				@ −0.7 V (vs. RHE)	
CO_2_RR	Pore Zn nanosheets	*fcc*	0.1 M KHCO_3_	90% FE for CO	[102]
				@ −1.0 V (vs. RHE)	
CO_2_RR	Hexagonal Zn nanoplates	*fcc*	0.5 M KHCO_3_	85.4% FE for CO	[103]
				@ −0.85 V (vs. RHE)	
CO_2_RR	Bismuthene	*fcc*	0.5 M KHCO_3_	∼100% FE for HCOOH	[104]
				@ −0.83 to –1.18 V (vs. RHE)	
CO_2_RR	Mesoporous Bi nanosheets	*fcc*	0.5 M NaHCO_3_	∼ 99% FE for HCOOH	[105]
				@ −0.9 V (vs. RHE)	
CO_2_RR	Bi nanosheets	*fcc*	0.5 M NaHCO_3_	95% FE for HCOOH	[106]
				@ −1.5 V (vs. SCE)	
CO_2_RR	Bismuthene	*fcc*	0.5 M KHCO_3_	∼ 99% FE for HCOOH	[107]
				@ −0.58 V (vs. RHE)	
CO_2_RR	Partially oxidized	*hcp*	0.1 M Na_2_SO_4_	90.1% FE for HCOOH	[29]
	Co nanosheets			@ −0.85 V (vs. RHE)	
CO_2_RR	Nanodefective Cu nanosheets	*fcc*	0.1 M K_2_SO_4_	83.2% FE for ethylene	[108]
				@ −1.18 V (vs. RHE)	

^a^Phosphate buffered saline; ^b^saturated calomel electrode.

### Hydrogen evolution reaction

HER is a key half reaction for producing hydrogen via electrochemical water splitting. 2D noble metal nanomaterials, e.g. Pt [23], Pd [36], Ir [55], Rh [67,68] and Ru [89], possess moderate hydrogen adsorption and desorption properties, thus showing superior catalytic performance in HER. For example, 2D Pt nanodendrites with a thickness of ∼2.3 nm exhibited extremely low overpotential (<10 mV) at a current density of 50 mA cm^−2^ in 0.5 M H_2_SO_4_, while the commercial Pt/C required 12.5 mV to achieve the same current density [23]. The enhanced performance of HER can be explained as follows. First, the dendritic morphology exposes more catalytic active sites in both corners and edges of the branches. Second, the single-crystalline nature leads to faster electron and mass transfer on 2D Pt nanodendrites. Third, the ultrathin 2D nanodendrite structure can not only increase the utilization of Pt atoms to improve HER activity, but also avoid the Ostwald ripening of the nanodendrites during electrocatalysis to ensure catalytic stability. Cheng *et al.* demonstrated that ultrathin Ir nanosheets outperformed Pt/C in both alkaline and acidic conditions due to their ultrathin 2D morphology and partially hydroxylated surfaces. Impressively, HER activity can be further improved by synthesizing 2D noble metal alloy nanomaterials with finely tuned electronic structures. For example, 2D IrRh [40] and RuRh [41] nanosheets are regarded as ideal electrocatalysts for HER.

Due to the scarce nature of noble metals, alloying 2D noble metal nanomaterials with non-noble metals is considered a cost-effective strategy [30,64,66]. Recently, our group prepared RuNi alloy nanostructures that were composed of multilayered nanosheets, achieving a low overpotential of 15 mV at 10 mA cm^−2^ (η_10_) and a small Tafel slope of 28 mV dec^−1^ in 1.0 M potassium hydroxide (KOH). This surpasses commercial Pt/C and Ru/C [64]. The enhanced HER performance was ascribed to the large electrochemically active surface area (ECSA) arising from the 2D structure and the electronic effect from Ni alloying.

**Figure 5.**
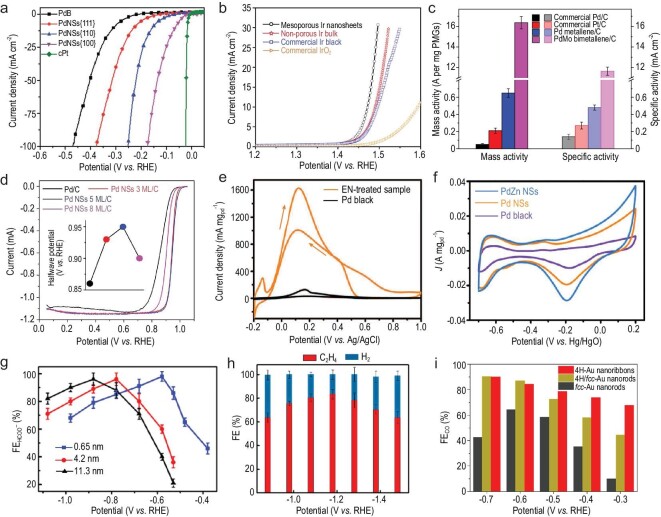
Electrocatalytic performance of 2D metal nanomaterials. (a) HER polarization curves of Pd nanosheets with exposed facets of {100}, {110} and {111}, Pd black (PdB) and commercial Pt (cPt) in 0.5 M H_2_SO_4_ at a scan rate of 5 mV s^−1^. Reproduced with permission from ref. [36]. Copyright 2018 the Royal Society of Chemistry. (b) OER polarization curves of mesoporous Ir nanosheets, nonporous Ir bulk, commercial Ir black and IrO_2_ catalysts in 0.5 M H_2_SO_4_ at a scan rate of 5 mV s^−1^. Reproduced with permission from ref. [54]. Copyright 2018 American Chemical Society. (c) Comparison of the mass and specific ORR activities of PdMo bimetallene/C, Pd metallene/C and the commercial Pt/C and Pd/C catalysts in 0.1 M KOH at 0.9 V (vs. RHE). Reproduced with permission from ref. [60]. Copyright 2019 Nature Publishing Group. (d) ORR polarization curves of Pd nanosheets with average thicknesses of three monolayers, five monolayers and eight monolayers, respectively, in 0.1 M KOH. Inset: halfwave potential of Pd nanosheets. Reproduced with permission from ref. [48]. Copyright 2019 American Association for the Advancement of Science. (e) Cyclic voltammetry (CV) curves of EN‐treated PdCu nanosheets and Pd black in FAOR recorded in the aqueous solution containing 0.5 M H_2_SO_4_ and 0.25 M HCOOH at a scan rate of 50 mV s^−1^. Reproduced with permission from ref. [63]. Copyright 2017 John Wiley & Sons, Inc. (f) CV curves of PdZn nanosheets, Pd nanosheets and Pd black in EOR test in a mixture of 1.0 M NaOH and 1.0 M ethanol at a scan rate of 50 mV s^−1^. Reproduced with permission from ref. [58]. Copyright 2019 American Chemical Society. (g) Thickness-dependent Faradaic efficiency for HCOO^−^(FE_HCOO^−^_) on Bi nanosheets. Reproduced with permission from ref. [107]. Copyright 2020 Nature Publishing Group. (h) FEs versus applied potentials for CO_2_ electroreduction on defected Cu nanosheets. Reproduced with permission from ref. [108]. Copyright 2020 American Chemical Society. (i) Faradaic efficiency for CO (FE_CO_) of Au nanoribbons, 4H/*fcc* nanorods and *fcc* nanorods for CO_2_RR. Reproduced with permission from ref. [109]. Copyright 2020 American Chemical Society.

To reduce the use of noble metals, 2D non-noble metal alloy electrocatalysts have been prepared and used for HER [92,93]. For instance, in the alkaline condition, the ultrathin NiMo nanosheet displayed faster electron and mass transfer rate compared to the commercial Pt/C, due to its ultrathin 2D morphology and finely tuned alloy composition [92]. As is known, defects like vacancies can be more easily generated in 2D metal nanomaterials since the surface atoms are more likely to escape from the 2D lattice as the thickness decreases [11]. The hierarchical Rh nanosheets with ordered vacancies prepared recently exhibited a lowest η_10_ of 37.8 mV compared with those of Rh/C (58.7 mV) and Pt/C (66.0 mV) in alkaline media. The enhanced HER performance of hierarchical Rh nanosheets was mainly attributed to the unique VBP structure and ultrathin 2D sheet-like morphology [68]. In addition, using 2D Pd nanosheets with different exposed facets as electrocatalysts, Xu *et al.* investigated the facet-dependent HER performance [36]. Owing to the optimal balance between adsorption and desorption of hydrogen on Pd(100) planes, Pd nanosheets exposed with the (100) planes showed a lowest η_10_ of only 67 mV in acidic electrolyte, compared to those with exposed (110) planes (158 mV) and (111) planes (227 mV) (Fig. 5a). Moreover, the performance of Pd nanosheets with (100) planes only exhibited a slight degradation over 20 h. This superior HER durability could be attributed to the anisotropic ultrathin 2D structure, which greatly inhibited the dissolution and ripening process of Pd nanosheets.

2D noble metal nanomaterials can exhibit remarkable HER performance through the selective exposure of specific facets or control of their thickness. In addition, alloying with non-noble metals is a cost-effective strategy for 2D noble metal nanomaterials used for HER.

### Oxygen evolution reaction

As the anodic half reaction of overall water splitting, OER plays a significant role in proton exchange membrane water electrolyzers. However, the sluggish kinetics of the multistep proton-coupled electron transfer process in OER has seriously impeded the overall efficiency of water splitting [9]. To date, Ir-based materials have been regarded as the most promising electrocatalysts towards OER due to their superior catalytic property [54,55,94]. Recently, the preparation of 2D Ir-based electrocatalysts for OERs has been extensively reported. For instance, the ultrathin mesoporous Ir nanosheets synthesized by Jiang *et al.* displayed superior OER activity in 0.5 M H_2_SO_4_, achieving η_10_ of 240 mV, which was smaller than the non-porous Ir bulk (260 mV), commercial Ir black (268 mV) and commercial IrO_2_ (360 mV) (Fig. 5b) [54]. The outstanding OER performance of the mesoporous Ir nanosheets could be ascribed to the unique electronic states of 2D structures as well as the large exposed surface area and rich active sites on the mesoporous structure. In addition, the partially hydroxylated ultrathin Ir nanosheets also exhibited improved OER activity in a wide pH range, achieving η_10_ of 328 and 266 mV in 0.5 M H_2_SO_4_ and 1 M KOH, respectively, which were both lower than those of the commercial IrO_2_ [55]. Impressively, Ir-based bimetallic alloy (IrRh) nanosheets could also be considered as catalysts for OER in pH-universal electrolytes, and further applied to overall water splitting [40]. Additionally, other 2D bimetallic alloys, for instance, the channel-rich RuCu nanosheets, reached small η_10_ of 234, 276, 236 and 240 mV in 1 M KOH, 0.1 M KOH, 0.5 M H_2_SO_4_ and 0.05 M H_2_SO_4_, respectively, exhibiting excellent OER catalytic activity [30]. For the overall water splitting, the RuCu nanosheets with optimized electronic structures displayed pH-universal water-splitting performance, achieving η_10_ at a cell voltage of 1.49, 1.55, 1.49 and 1.50 V in 1 M KOH, 0.1 M KOH, 0.5 M H_2_SO_4_ and 0.05 M H_2_SO_4_, respectively; much lower than those of the commercial Ir/C || Pt/C.

State-of-the-art OER catalysts are mainly based on noble metal catalysts. 2D noble metal catalysts can exhibit high utilization of noble metal atoms and superior intrinsic activity due to their 2D structure, thus representing a promising candidate for future high-performance OER catalysts.

### Oxygen reduction reaction

ORR is the cathodic half reaction operated in fuel cells. However, the commercial application of proton exchange membrane fuel cells has been severely hindered by the sluggish kinetics of current ORR catalysts [7,9]. Although the state-of-the-art Pt-based catalysts are still the most commonly used ORR catalysts owing to their high efficiency, the extensive application of Pt-based catalysts generally suffers from the high price of Pt [95]. Therefore, preparation of Pt-based catalysts with 2D morphology is an efficient strategy for increasing the atom utilization of Pt, and thus improving ORR activity. Owing to the high specific surface area enlarged by the porous structure and the abundant active sites offered by numerous defects, the ultrathin porous Pt nanosheets synthesized by Feng *et al.* displayed excellent ORR performance with specific activity of 3.1 mA cm^−2^ and mass activity of 2.07 A mg_Pt_^−1^ at 0.90 V versus the reversible hydrogen electrode (vs. RHE) in 0.1 M HClO_4_, which were 10.7 and 9.8 times those of the commercial Pt/C, respectively [96]. Importantly, the synthesized ultrathin porous Pt nanosheets also displayed superb durability, enduring 30 000 cycles of accelerated durability testing without obvious activity decay. Moreover, the same group further prepared hexagonal PtBi nanoplates as efficient ORR catalysts, showing extraordinary tolerance to CH_3_OH, HCOOH and CO [56]. The PtBi nanoplates displayed a high mass activity of 1.04 A mg_Pt_^−1^ (vs. RHE) in 0.1 M HClO_4_, which was higher than those of the recently reported non-2D PtBi nanomaterials. Besides, the 2D Pd-based catalysts are considered as alternatives to Pt [48,60,80]. For example, Jiang *et al.* synthesized SAL PdCo alloy nanosheets as high-performance ORR catalysts [80]. The PdCo SAL nanosheets showed a high mass activity of 0.955 A mg_Pd_^−1^ at 0.9 V (vs. RHE) in 0.1 M HClO_4_, which was 3.3, 6 and 33 times those of Pd SAL (0.289 A mg_Pd_^−1^), commercial Pt nanoparticle (0.159 A mg_Pt_^−1^) and Pd nanoparticle (0.029 A mg_Pd_^−1^), respectively. The enhancement of ORR performance could be ascribed to the ultrathin SAL structure, achieving nearly full atom utilization. Remarkably, the PdMo nanosheet prepared by Luo *et al.* was a highly efficient electrocatalyst for ORR in alkaline media, overcoming the difficulty for Pt-based catalysts to achieve optimized oxygen binding strength in the presence of hydroxides [60]. Due to the large ECSA (138.7 m^2^ g_Pd_^−1^) of the ultrathin 2D structure, the PdMo nanosheets manifested an outstanding ORR mass activity of 16.37 A mg_Pt_^−1^ at 0.9 V (vs. RHE) in 0.1 M KOH (Fig. 5c).

Impressively, the ORR performance of 2D metal electrocatalysts can be finely tuned by changing their thickness. For example, Wang *et al.* demonstrated the thickness-dependent ORR performance of Pd nanosheets [48]. Evidenced by the computational and experimental results, Pd nanosheets with a thickness of 5 ± 1 monolayers exhibited better ORR performance compared to Pd nanosheets with 3 ± 1 monolayers and 8 ± 1 monolayers in both acidic and alkaline media (Fig. 5d). The good performance of Pd nanosheets with 5 ± 1 monolayers originated from the optimal surface strain, allowing suitable binding with reaction intermediates on the catalyst surface.

2D noble metal electrocatalysts show intriguing ORR activity, which is related to their thickness. 2D metal nanomaterials with single- or few-atomic layers expose rich under-coordinated atoms at the surface, which have been recognized as highly active centers for ORR. Next generation 2D noble metal-based catalysts should focus on the fine control of their thickness, as well as the improvement of their stability.

### Chemical fuel oxidation reaction (FAOR, MOR and EOR)

Organic acids and alcohols, such as formic acid, methanol and ethanol, are commonly used chemical fuels in direct liquid fuel cells [2,7]. 2D metal nanomaterials have been regarded as excellent electrocatalysts for the oxidation reaction of these chemical fuels, owing to their large exposed surface area and abundant active sites. Notably, 2D Pd-based nanostructures are outstanding electrocatalysts for FAOR due to their high catalytic activity and excellent anti-poisoning capacities [24,62,97,98]. Recently, Qiu *et al.* prepared the self-assembled porous Pd nanosheets as efficient and stable electrocatalysts for FAOR [88]. Since the ultrathin knit-like 2D morphology and porous structure provided rich active sites and a high specific surface area, the Pd nanosheets showed higher catalytic activity, superior durability and better anti-poisoning capability compared to the commercial Pd black catalyst. Our group reported that the FAOR activity could be significantly enhanced by alloying Pd with Cu [63]. The ultrathin PdCu nanosheets showed a superior mass activity of 1655.7 ± 74.6 mA mg_Pd_^−1^ in an acidic electrolyte, due to the ultrathin 2D morphology, optimized electronic structure, synergistic effect between Pd and Cu, and post-treatment with ethylenediamine (EN) (Fig. 5e).

Besides FAOR, 2D metal nanomaterials, e.g. Pd [34,58,61,99] and Rh [83], have been considered as promising Pt-alternative electrocatalysts for MOR and EOR owing to their excellent catalytic activity and stability. For instance, Zhu *et al.* reported hierarchical porous Rh nanosheets as efficient electrocatalysts for MOR [83]. The rich grain boundaries and ultrathin porous structure endowed the hierarchical porous Rh nanosheets with a mass activity of 333 A g^−1^ at 0.63 V (vs. RHE) in alkaline electrolyte, much higher than that of the commercial Pt black catalysts (57.4 A g^−1^). Ge *et al.* also used ultrathin Pd nanomeshes as an electrocatalyst for EOR. These exhibited high mass activity of 5.40 A mg _Pd_^−1^ and specific activity of 7.09 mA cm^−2^ in alkaline media due to their unique mesoporous structure and large surface area [49]. Additionally, 2D Pd-based bimetallic alloy nanostructures have also been demonstrated to be good electrocatalysts for MOR and EOR, e.g. PtPb [99] and PdCu [61], used for MOR, and PdAg [34] and PdZn [58], used for EOR. For instance, PdZn nanosheets reported by our group displayed enhanced EOR activity, with a mass activity of 2.73 A mg_Pd_^−1^ in a mixture of 1.0 M NaOH and 1.0 M ethanol, which was 1.74 and 2.97 times those of the pure Pd nanosheets (1.57 A mg_Pd_^−1^) and commercial Pd black (0.92 A mg_Pd_^−1^), respectively (Fig. 5f) [58]. The enhanced EOR activity of PdZn nanosheets can be explained as follows. First, the 2D morphology of PdZn nanosheets provided a large surface area and rich uncoordinated active sites for the electrochemical EOR. Second, the introduction of Zn into Pd tuned the electronic structure of Pd, thereby improving the EOR activity. Third, lattice defects (e.g. dislocations and grain boundaries) in polycrystalline structures also altered the electronic structure, and offered extra active sites for EOR [100].

Nowadays, alloying 2D noble metal nanomaterials with other non-noble metals is a promising strategy to enrich the library of cost-effective 2D bi- or multi-metallic alloy electrocatalysts for FAOR, MOR and EOR. The introduction of other metals could optimize the electronic structures of 2D metal electrocatalysts, and the synergistic effect between multiple elements could further improve catalytic performance.

### CO_2_ reduction reaction

CO_2_RR is one of the most important strategies for solving the global energy crisis caused by excessive consumption of fossil fuels, as it converts CO_2_ into high value-added products, e.g. CO, methane, methanol, formate, ethylene and ethanol [3]. 2D noble metal electrocatalysts exhibit promising application to CO_2_RR owing to their excellent selectivity, catalytic activity and stability [25]. For example, Liu *et al.* demonstrated that triangular Ag nanoplates could selectively reduce CO_2_ to CO in 0.1 M KHCO_3_, and possessed an enhanced Faradaic efficiency (FE) of 96.8% and excellent durability of 7 days [101]. Until now, 2D non-noble metal-based electrocatalysts for CO_2_RR, such as Zn [102,103], Bi [104–107], Co [29] and Cu [108], have attracted increasing research interest because of their cost-effectiveness compared to noble metals. For instance, Liu *et al.* used porous Zn nanosheets as the CO_2_RR electrocatalyst to selectively produce CO. The porous Zn nanosheets exhibited a high FE of 90%, large partial current density and outstanding durability for over 24 h in 0.1 M KHCO_3_, benefiting from 2D morphology and porous architecture with increased exposure and active sites [102]. In addition, Bi-based nanosheets have considerable potential to produce formate [104–107]. Owing to the enlarged surface area and rich under-coordinated Bi sites, the ultrathin Bi nanosheets exhibited a large catalytic current density (24 mA cm^−2^ at −1.74 V), excellent formate selectivity (FE of >90% over a broad potential range) and superior durability (>10 h) in 0.5 M NaHCO_3_, unveiling great potential with regard to electrocatalytic conversion from CO_2_ to formate [106]. Yang *et al.* studied the thickness-dependent CO_2_RR performance of Bi nanosheets (Fig. 5g). The monolayered Bi nanosheets with an average thickness of 0.65 nm exhibited higher FE of ∼99% and lower onset overpotential of <90 mV compared to those with a thickness of 4.2 nm and 11.3 nm, respectively. Moreover, during the CO_2_RR, the 2D atomic Co layers could also selectively and efficiently produce formate [29]. Interestingly, Cu-based nanomaterials have attracted tremendous attention due to their unique ability to convert CO_2_ or CO into high value-added multicarbon (C_2+_) products, paving the way for the sustainable production of fuels and chemicals. Zhang *et al.* demonstrated the nanodefective Cu nanosheets for the electrochemical CO_2_ reduction, which exhibited an ethylene FE of 83.2% with a current density of ∼60 mA cm^−2^ (Fig. 5h) [108]. Remarkably, 2D metal nanostructures with unconventional crystal phases may display enhanced CO_2_RR performance, since the combination of unconventional crystal phase and 2D morphology can result in the preferential exposure of under-coordinated sites. Au nanoribbons with the unconventional 4H phase have achieved >90% FE toward CO at −0.7 V (vs. RHE) in 0.1 M KHCO_3_, exhibiting better activity and selectivity for CO production, compared with the 4H/*fcc* nanorods and conventional *fcc* nanorods (Fig. 5i) [109]. Confirmed by theoretical calculations, the relatively high CO_2_RR activity of 4H nanoribbons could be attributed to the highly active and abundant 7- and 8-fold under-coordinated sites on the specific surfaces of 4H nanoribbons, i.e. the (11$\bar{2}$0)_4H_ and (1$\bar{1}$00)_4H_ ridge sites.

2D metal nanomaterials exposing well-defined specific facets favor the selective production of desired products such as CO, formate, and importantly, the C_2+_ products. Notably, 2D Cu nanomaterials with unconventional crystal phase or phase boundaries are more favorable for C–C coupling due to the proper ^*^CO adsorption energy, yielding a series of C_2+_ products, such as ethylene, ethanol and acetate.

## CONCLUSION AND PERSPECTIVES

With the rapid development of synthetic techniques of nanomaterials, recent decades have witnessed remarkable progress in the preparation of 2D metal nanomaterials with unique physicochemical properties. 2D metal nanomaterials with structural features like shape, thickness, size, composition and crystal phase have been successfully synthesized via various wet-chemical synthetic methods. Wet-chemical synthesis can achieve massive production of 2D metal electrocatalysts with finely controlled structures and abundant surface function groups, and could enhance the performance of electrocatalysis. To date, a large quantity of research work has been devoted to preparing 2D noble metal catalysts for various electrocatalytic reactions due to their superior intrinsic catalytic activity. Recently, alloying non-noble metals with noble metals and preparing 2D non-noble metals have attracted ever-growing research interest, as these strategies can not only reduce the catalyst cost, but also boost catalytic performance. Benefiting from the large exposed surface area and abundant active sites, the ultrathin 2D metal nanomaterials exhibit superior electrocatalytic performance in various reactions, including HER, OER, ORR, FAOR, MOR, EOR and CO_2_RR. Although tremendous efforts have been devoted to the preparation of 2D metal electrocatalysts, many challenges still remain. Based on the current research progress, some challenges and potential research directions are proposed in order to inspire more fascinating research work in the near future.

- **Investigating the formation mechanisms of 2D metal nanomaterials by *in situ* characterizations.** Despite the recent great progress made in the wet-chemical synthesis of numerous 2D metal nanomaterials, the formation mechanisms of these unique 2D metal nanostructures still need to be fully understood. Nowadays, most studies on the formation of 2D metal nanostructures are based on *ex situ* characterization techniques, such as TEM and X-ray diffraction (XRD), which are used to characterize the intermediate at a certain reaction time interval. Thanks to the rapid development of advanced *in situ* characterization techniques, by using the *in situ* TEM, Raman spectrum, XRD and synchrotron radiation spectrum, the formation mechanisms could be unraveled. For example, *in situ* synchrotron small-angle X-ray scattering (*in situ*-SAXS) can be used to reveal the formation kinetics, i.e. nucleation and subsequent growth of 2D metal nanomaterials through observing the emergence of nuclei, growth of nanocrystals and reconstruction of 2D morphology. Moreover, *in situ* X-ray absorption fine structure (*in situ*-XAFS) can provide additional information on the structural evolution of 2D metal nanomaterials, including the coordination configuration and oxidation state during their nucleation and subsequent growth processes. Importantly, by combining with theoretical calculations and simulations, a comprehensive understanding on the formation mechanisms of 2D metal nanomaterials could be realized.
- **Enriching the library of 2D metal nanomaterials.** Until now, in the periodic table of elements, less than half of the metals with 2D features have been reported. Therefore, there is still plenty of room to enrich the library of 2D metal nanomaterials. In particular, it is urgently desired to develop general synthetic methods to prepare 2D multi-metallic alloy nanostructures with tunable compositions, e.g. 2D high-entropy alloys (HEAs). Benefiting from the synergistic effect among multiple elements and multi-active sites of HEAs, the 2D HEA nanostructures may exhibit outstanding electrocatalytic performance. Therefore, through rationally tuning the elemental compositions of metal alloys, the library of 2D metal alloy nanomaterials will be greatly enriched. Impressively, high-throughput theoretical calculations, guided by rising artificial intelligence (AI) technology, e.g. machine learning algorithms, can be used to predict novel 2D functional metal and alloy nanomaterials with highly efficient electrocatalytic performance, which could be synthesized in the near future.
- **Finely tailoring the structures of 2D metal nanomaterials.** In order to develop electrocatalysts with enhanced performance, rational design and synthesis of 2D metal nanomaterials with finely tuned structural parameters, such as thickness, exposed specific facet, defect and crystal phase, are imperative. The first research direction is to finely tune the thickness of various 2D metal nanomaterials. For example, the controlled synthesis of 2D metal nanosheets with sub-nanometer thickness or even single layer is very intriguing yet challenging. The unique coordination environment in the aforementioned 2D metal nanosheets can efficiently affect their electronic structures, resulting in the enhancement of their electrocatalytic properties. Second, facet engineering has roused extensive interest in recent years. Therefore, selectively synthesizing 2D metal nanomaterials with specific exposed facets is an attractive research direction. Until now, through wet-chemical synthesis, 2D metal nanomaterials only exposed limited facets, e.g. {111} planes for *fcc* Rh and {0001} planes for *hcp* Ru. Hence, the synthesis of 2D metal nanostructures with other facets, especially high-index ones, is another research topic. The exposed high-index facets possess a high density of low-coordinated atoms, edges, steps and kinks, serving as active sites for electrocatalysis, and thus showing enhanced electrocatalytic performances compared with the conventional low-index facets such as {111} and {100} planes. Third, defect engineering has also been recognized as an effective approach to designing efficient electrocatalysts. Construction of abundant defects, such as dislocations, boundaries and vacancies, in the 2D metal nanomaterials can tune the electronic structures and create more active sites, thereby enhancing the electrocatalytic performance. Last but not least, phase engineering of nanomaterials (PEN) opens a new route towards the synthesis of 2D metal nanostructures with unconventional phases, i.e. unconventional crystal phase, amorphous phase and heterophase nanomaterials for various promising applications [110]. To date, although a few 2D metal nanostructures with unconventional phases have been successfully synthesized, they are just the tip of the iceberg. A large number of unknown 2D metal nanostructures with unconventional phases still need to be explored.
